# Integration and potential of teaching communication skills in the study of veterinary medicine in Germany

**DOI:** 10.3205/zma001449

**Published:** 2021-03-15

**Authors:** Alina Pohl, Luise Grace Klass, Christin Kleinsorgen, Dora Bernigau, Birte Pfeiffer-Morhenn, Stefan Arnhold, Marc Dilly, Christina Beitz-Radzio, Sandra Wissing, Lena Vogt, Mahtab Bahramsoltani

**Affiliations:** 1 Freie Universität Berlin, Department of Veterinary Medicine, Berlin, Germany; 2 University of Veterinary Medicine Hannover, Foundation, Hannover, Germany; 3 Leipzig University, Faculty of Veterinary Medicine, Leipzig, Germany; 4 Justus-Liebig-University Giessen, Faculty of Veterinary Medicine, Giessen, Germany; 5 scil vet acadamy, Viernheim, Germany; 6 Ludwig-Maximilians-Universität Munich, Faculty of Veterinary Medicine, Munich, Germany

**Keywords:** veterinary medical education, communication skills, soft skills, curriculum development

## Abstract

**Goal: **Presentation of the current range of courses regarding communication at the five German educational institutions for veterinary medicine. In addition to learning objectives and individual solutions, possible potential for future developments are presented.

**Methods: **Interviews with communication educators at the five German education institutions and subsequent synopsis.

**Results: **To date, there are no binding education guidelines regarding communication in veterinary medicine. Nevertheless, communication education has been introduced at all five education institutions, albeit depth and formats vary considerably. The learning objectives are largely consistent and based on the recommendations for day-one-skills made by the European Association of Establishments for Veterinary Education. Communication is not recognized as a fully-fledged subject in the curricula of any of the education institutions. All education institutions clearly fall short of teaching the recommended 150 lecture hours.

**Conclusion: **To ensure communication skills in veterinary medicine graduates, binding education guidelines should be agreed upon. Communication education should be integrated into all veterinary curricula as a fully-fledged subject with longitudinally increasing depth.

## Zusammenfassung

**Zielsetzung: **Darstellung des aktuellen Lehrangebots im Bereich Kommunikation an den fünf deutschen veterinärmedizinischen Bildungsstätten. Neben Lernzielen und individuellen Lösungswegen wird auch das mögliche Entwicklungspotential gezeigt.

**Methodik:** Befragung der Kommunikationslehrenden an den fünf deutschen Bildungsstätten und anschließende Zusammenfassung.

**Ergebnisse: **Bisher gibt es keine rechtlich bindenden Ausbildungsvorgaben für Kommunikation in der Veterinärmedizin. Trotzdem erfolgt Kommunikationslehre mittlerweile an allen fünf Bildungsstätten, wobei sich Umfang und Formate deutlich voneinander unterscheiden. Die Lernziele sind überwiegend deckungsgleich und orientieren sich an den Empfehlungen für Day-One-Skills der European Association of Establishments for Veterinary Education. Kommunikation ist an keiner der Bildungsstätten als vollwertiges Fach im Curriculum integriert. Der empfohlene Umfang von 150 Stunden wird an allen Bildungsstätten deutlich unterschritten.

**Schlussfolgerung:** Zur Sicherung der Kommunikationskompetenz veterinärmedizinischer Absolvent*innen sollten bindende Vorgaben für die Ausbildung beschlossen werden. Die Kommunikationslehre sollte als vollwertiges Fach in alle Curricula integriert werden und außerdem longitudinal steigernd konzipiert sein.

## Introduction

Early development of communication skills is a key component to professional success in all veterinary fields [6]. However, communication education is not even mentioned in the Federal Ordinance for Licensing of Veterinarians in Germany (TAppV) [https://www.gesetze-im-internet.de/tappv/index.html#BJNR182700006BJNE000100000]. Nevertheless, the importance has been recognized on a European level. The European Association of Establishments for Veterinary Education (EAEVE) lists all competences that veterinary students should obtain throughout their education in their “Day-One-Skills” [13]. Specific recommendations for communication skills have been listed among them for some time. Ideally, veterinarians should be able to communicate with various parties effectively. Aside from clients, they must be capable of communicating with employees, colleagues, business partners and the general public. Moreover, they must be able to listen actively and provide relevant information to a specific audience in a comprehensible manner. 

As part of the “SOFTVETS”-project, ten specific fields that veterinary students should obtain communication skills in, were devised [27]. In addition to the aforementioned competences, they explicitly list verbal, non-verbal and paraverbal communication as well as structuring conversations. Veterinarians must also be capable of showing empathy, evince own weaknesses and limitations as well as reflect on their own communication, to actively shape it. Furthermore, veterinarians must be able to recognize sensitive topics and act appropriately. In turn, the necessary competences lay the basis for achieving these learning goals [7], [13], [26], [28]. However, German education institutions are yet to publish uniform learning objectives [32]. 

The demands of the veterinary profession result in a duty to implement communication education in the veterinary curriculum, which is also a requirement for successful accreditation of the veterinary education institutions. Since specific communication skills are not yet an explicitly named requirement [13], veterinary education institutions that imply communication teaching have been accredited. 

Nevertheless, some European countries have already established (longitudinal) communication education [20], [23], [34], [39]. Communication is taught at almost all veterinary education institutions throughout the United Kingdom and North America, as required by their accreditation rules [2], [20], [34]. Moreover, communication has been named as a professional competence by the Roadmap North American Veterinary Medical Education Consortium (NAVMEC) [1]. Many studies have investigated this issue and underline the importance for the acquisition of communication skills for veterinarians [24], [25], [30], [31], [37]. Professional success as a veterinarian greatly correlates with own communication skills [4]. Communication skills have significant influence on client satisfaction, therapy compliance, the related consultation outcome and not least on the veterinarian’s job satisfaction [24], [37], [38]. Yet, communication education is not a compulsory part of veterinary education in Germany. Hence, the professional education of future veterinarians and accepting the significance of communication skills lies in the hands of the education institutions [28]. To embrace the responsibility for communication education in veterinary medicine in Germany, a pan-university working group was founded by the German Veterinary Society’s (DVG) specialist group for didactics and communication, the Committee for Veterinary Medicine of the Society for Medical Education (GMA) and the Competence Centre for E-Learning, Didactics and Education Research in Veterinary Medicine (KELDAT). The working group held an open workshop at the DVG Congress 2015, during which the first draft of a model curriculum for the longitudinal implementation of communication education was developed. The group concluded that three requirements needed to be met:

- explicit recognition of communication as a fully-fledged subject in the TAppV
- reduction or integration of other subjects to allow time to teach communication skills, and
- a continuously increased depth of teaching to enhance communication skills throughout the degree program.

Furthermore, a list of learning objectives was devised: Veterinarians should learn to foster a relationship with various conversation partners, structure a conversation, obtain information and present it comprehensibly. Moreover, they should be capable of giving and receiving feedback. In addition, they should know and be able to apply strategies to communicate effectively in difficult situations. Teaching the basics of communication was also included as a learning objective. The definitions and various levels of expression should be known, and students should be able to apply different models of communication. Furthermore, the necessary competences of communication educators were defined. Lastly, specific plans for the implementation of communication education, time scale, timing within the curriculum as well as suitable examination formats were discussed [26]. Since the TAppV has not been changed with regard to communication education since the workshop in 2015, the responsibility for this subject depends entirely on the education institutions or rather on motivated individuals among the veterinary lecturers. 

This article depicts the status quo on communication education at veterinary education institutions in Germany (Freie Universitaet Berlin, Justus-Liebig-Universitaet Giessen, University of Veterinary Medicine Hannover, Foundation, Leipzig University, Ludwig-Maximilians-Universitaet Munich). In addition to learning objectives and individual solutions, the potential for further developments are elaborated. 

## Method

A three-step process was used to obtain an overview of the status quo of communication education at all five veterinary education institutions. First, communication educators from all institutions were asked if they were interested in participating as an author in this article. Those known personally to the initiators were contacted via telephone and invited to include additional communication educators as potential authors. Next, all authors filled out a qualitative questionnaire, which included the following questions regarding communication education at their institution:

- Which teaching formats are used?
- What content is taught using these teaching formats?
- What are the learning objectives?
- Who participates in the teaching formats?
- How many hours are taught?
- Is there an examination? If so, what format is used (summative/formative)?

The results of this study were then summarized in a table. Finally, authors discussed future perspectives in a written exchange and summarized the consequent consensus. 

## Results

The summarized overview of teaching formats, course content and learning goals at the five German veterinary education institutions in attachment 1 shows that, while the formats and extent of communication education vary significantly, the learning objectives are largely the same (see table 1 (Tab. 1), table 2 (Tab. 2), table 3 (Tab. 3), table 4 (Tab. 4), table 5 (Tab. 5)). All education institutions have integrated communication education as best they can, given their respective structure and existing system. In doing so, the initiation and establishment of teaching formats beyond extracurricular classes was achieved to varying degrees through third-party funded projects at most education institutions. Nevertheless, communication skills have been and are implicitly taught, although communication education has not been explicitly included, for instance through observational learning [41] and are still a part of other examinations [10]. The TAppV enables veterinary education institutions to design and test their own curricula or practice their own interpretation of the education regulations to improve veterinary medical education (§3 Trial clause [Erprobungsklausel]). Based on this, lecture hours of existing classes were reallocated in favour of communication education. In Berlin, Giessen and Hannover, basic theoretical education is compulsory for all students in the first or second semester (see attachment 1 and table 1 (Tab. 1), table 2 (Tab. 2), table 3 (Tab. 3)). For this purpose, four hours of the lecture series veterinary professional studies were dedicated to communication education in Giessen and Hannover. These veterinary professional studies class in Leipzig, however, has been used to improve business administration skills. In Berlin, a blended-learning course is used to provide the theoretical basis. The course was developed as part of the restructuring of interdisciplinary lectures, which was achieved through the QuerVet project [12]. Hence, the hours for this compulsory class originated from interdisciplinary lectures. According to the TAppV, students should take a total of 196 hours of interdisciplinary lectures over the course of multiple semesters. They are meant to be focused on interdisciplinary content and tasks relevant to clinical practice. Therefore, it became possible to implement one weekly lecture hour (0.5 ECTS) for communication education into the existing curriculum without increasing the number of compulsory lecture hours for students. Furthermore, students in Berlin, Giessen, Hannover and Munich have to exercise conversation skills in small groups as part of the practical year (semesters 9-10), and additionally as part of the clinical propaedeutics class in Giessen (see table 1 (Tab. 1), table 2 (Tab. 2), table 3 (Tab. 3) and table 5 (Tab. 5)). This training starts with a theoretical introduction and is followed by practicing conversation skills in different settings as well as group feedback. In addition, a large part of communication education at all five education institutions is offered through extracurricular classes with foci ranging from theoretical basics to practical conversation exercises for different situations and subsequent peer feedback. Furthermore, Hannover, Leipzig and Munich offer communication education as a skills lab station (see table 3 (Tab. 3), table 4 (Tab. 4), table 5 (Tab. 5)). The Communication Day is an additional voluntary educational offer to students in the fifth semester in Berlin, during a project week (see table 1 (Tab. 1)). After a brief theoretical input, students can practice their communication skills in various scenarios in small groups and receive peer feedback. 

To provide the theoretical basis for communication, all veterinary education institutions employ the Four-sides model by F. Schulz von Thun [36]. Furthermore, Berlin, Hannover, Leipzig and Munich use the transactional analysis by Berne [5], while Hannover and Munich use the Eisberg-model [16], Giessen and Munich include the Shannon-Weaver-model [33] and Giessen also uses Geisser’s communication model [21]. The Calgary-Cambridge Observation Guide is used to structure veterinary conversation in Berlin, Giessen, Hannover and Leipzig [19]. In Giessen and Munich, the conversation model by Rogers [40] and the 5-why-method [21] are used additionally. Dealing with difficult conversation settings is addressed through the SPIKES model in Giessen and Hannover [8]. In addition, the CALM model [35] is used in Giessen, while the WWSZ technique [29] is used in Hannover. No matter which of the various models for communication and conversation are applied, methods for giving and receiving feedback are trained through practical exercises at all five education institutions [14], [28].

Currently, trained lay people and professional actors are used to simulate conversation settings in Giessen, Hannover and Munich (see table 2 (Tab. 2), table 3 (Tab. 3) and table 5 (Tab. 5)). The actors are also included in a 360° feedback after completing the conversation simulation. 

As an additional examination format, students in Hannover are examined at a communication station through a checklist [11] as part of an electronic objective structured clinical examination (eOSCE) during their practical year at the Small Animal Hospital (see table 3 (Tab. 3)). An overview of the status quo of communication education at German veterinary education institutions is provided in attachment 1 . 

## Discussion

Communication plays a vital role in all veterinary fields and communication identity formation is a crucial process that should be encouraged during the course of study [15]. Ideally, communication education should include about 150 compulsory training hours. Based on this proposal for developing a model curriculum, communication education should include 30 hours during the preclinical semesters. Thereof, only 10 hours should focus on theoretical basics of communication, while 20 hours should be devoted to training how to give feedback in small groups and thereby require students to actively participate. During the clinical semesters, communication education should include 36 training hours to enhance conversation skills in veterinary-specific settings during interdisciplinary lectures and an additional 84 hours as part of clinical propaedeutics and the practical year, for students to practice and apply their knowledge to various clinical settings [26].

Obviously, none of the German veterinary education institutions have implemented or been able to implement this yet. While compulsory classes are taught in small groups during the clinical semesters as part of the practical year at many universities, the lecture hours are very limited. In addition, extracurricular classes are offered at all education institutions. This suggests that some educators recognize communication education as a crucial element of veterinary education and are very enthusiastic about creating appropriate learning opportunities for students. Aside from the range of extracurricular courses, all students should have an equal opportunity, which can only be achieved by providing courses that provide places for all interested students [26]. It has also been shown that students prefer extracurricular classes that have a low workload or prepare them for (examination) performance [9], which calls the current concept into question, as it is based entirely on voluntariness. 

The fact that communication education is yet to be recognized as a fully-fledged subject by the TAppV remains a challenge for the integration into the veterinary curriculum, since no department is officially responsible for the teaching content. As a result, communication education is shouldered by a few enthusiastic individuals and largely funded by third-party grants. Particularly small group teaching requires a substantial dedication of time and staff capacities. Since there is no official recognition of this subject, education institutions often fail to maintain the small number of lectures with appropriate competences to teach communication in the long term. Yet, this is an important requirement to ensure quality improvement processes and measures as well as secure continuity for students’ education. 

Currently, all veterinary education institutions are trying to integrate extracurricular competences into the subject-oriented curriculum, as best they can. Communication education at all five institutions is focused on the same learning objectives and covers the level of theoretical application, at least, through different teaching formats (see attachment 1 ). The Four-sides model is used for this purpose at all five institutions [36], while some education institutions use additional communication models [5], [16], [21], [33]. Regarding models for conversation and their application and transmission to different settings, there are some similarities in communication education for general veterinary conversations [19], although the education institutions set different foci in some regards (see attachment 1 ). For example, difficult conversations are only explicitly addressed in Giessen and Hannover [8], [29], [35]. Voluntary extracurricular classes are offered at all education institutions to train communication competences and voluntary or compulsory classes are part of practical training during clinical propaedeutics, the practical year and or a skills lab station. Seminars and small group classes are the predominant teaching formats for all classes regarding theoretical basics and simulated conversation practice. Furthermore, theoretical basics of communication are taught during compulsory classes in a lecture setting in Giessen and Hannover, and in a blended-learning format in Berlin. Additionally, an extracurricular e-learning class is offered to students in Hannover. Teaching formats that include theoretical input as well as its practical application with constructive peer-feedback and experienced teachers achieves excellent results regarding effectiveness [22]. On the one hand, this once again emphasizes the importance of explicitly implementing communication education into the curriculum. On the other hand, it demonstrates that this is an interesting prospective research field, which adds a research perspective to the teaching component of this field. Moreover, results of a study found that e-learning as well as role playing games and a combination of both methods can be used to improve communication competences of medical and educational science students. The combination of both methods showed the best results. However, the e-learning course that included the assessment of positive and negative examples had a higher learning effect compared to the role-playing games [17]. This result is supported by the concept of observational learning by Bandura [3]. The comparison of successful and unsuccessful behaviour has a very special learning effect on a cognitive level [18]. This realization is particularly relevant, since the number of participants in e-learning formats is unlimited, which could help realize compulsory classes for large numbers of students. Moreover, this interfaces with digital competences, another field in need of increased recognition as an essential part of veterinary education. Finally, teaching social and business administration skills also remain challenging. 

## Current professional roles of the authors

- Dr. Alina Pohl is a post-doc researcher at the Veterinary Hospital for Reproduction. She teaches discipline-specific topics of reproduction medicine, takes part in clinical work and emergency services. She researches acute metritis in dairy cows and dairy farm training. She is also actively involved in shaping and implementing communication education.
- Luise Grace Klass is a senior veterinary student and student assistant at the Institute of Veterinary Anatomy at Freie Universitaet Berlin. She supports communication education and education research projects. Furthermore, she advocates student-centred education in veterinary medicine as president of the student body.
- Dr. Christin Kleinsorgen is a post-doc researcher in E-Learning-Consulting. She develops and consults on creating digital and learning-theory-based teaching and learning materials, teaches communication and researches in the field of e-learning as well as key-competence training. She is actively involved in developing and implementing communication education in the clinical skills lab.
- Dr. Dora Bernigau is a post-doc researcher at the Institute of Veterinary Anatomy at the Faculty of Veterinary Medicine at Leipzig University. She teaches an elective communication class and has been in charge of establishing electronic examinations and teaching evaluations since 2018.
- Dr. Birte Pfeiffer-Morhenn is a post-doc researcher for teaching innovation at the Office of Student Affairs of the Faculty of Veterinary Medicine at JLU Giessen.
- Prof. Dr. Stefan Arnhold has been a professor at the Institute of Veterinary Anatomy, Histology and Embryology since 2007 and academic dean of the Faculty of Veterinary Medicine since 2019, and previously from 2010 to 2016.
- Dr. Marc Dilly is coordinator of advanced and continuing education of a global trading company for veterinary diagnostics. He has been teaching communication in veterinary medicine since 2013. He has been a visiting lecturer at the Faculty of Veterinary Medicine at JLU Giessen since 2016.
- Dr. Christina Beitz-Radzio is a post-doc at the Office of Student Affairs at the Faculty of Veterinary Medicine at LMU Munich. She is an instructor for tutors (TutorPlus) and a communication trainer (Sprachraum eG).
- Dr. Sandra Wissing is a post-doc researcher at the Clinical Skills Lab of the University of Veterinary Medicine Hannover, Foundation. She is responsible for the conceptualization, implementation and conduction of teaching communication, including the provision of teaching materials and coordinating classes with actors in the Clinical Skills Lab.
- Lena Vogt is a doctoral candidate in the QuerVet-project and staff member of the mentoring project, which organizes the Communication Day. She is involved in improving interdisciplinary classes by implementing virtual patients and working in cooperation with department specialists to integrate extracurricular skills into veterinary medical education.
- Prof. Dr. Mahtab Bahramsoltani is a professor at the Institute of Veterinary Anatomy. Her research is focussed on veterinary education and includes projects on test anxiety and self-efficacy expectations of veterinary students. She is engaged in the continuing education of veterinarians regarding communication and is actively involved in shaping and implementing communication education for veterinary students.

## Competing interests

The authors declare that they have no competing interests. 

## Supplementary Material

Communication education at the five German veterinary education institutions; results from a written questionnaire among teachers from the respective institutions

## Figures and Tables

**Table 1:**
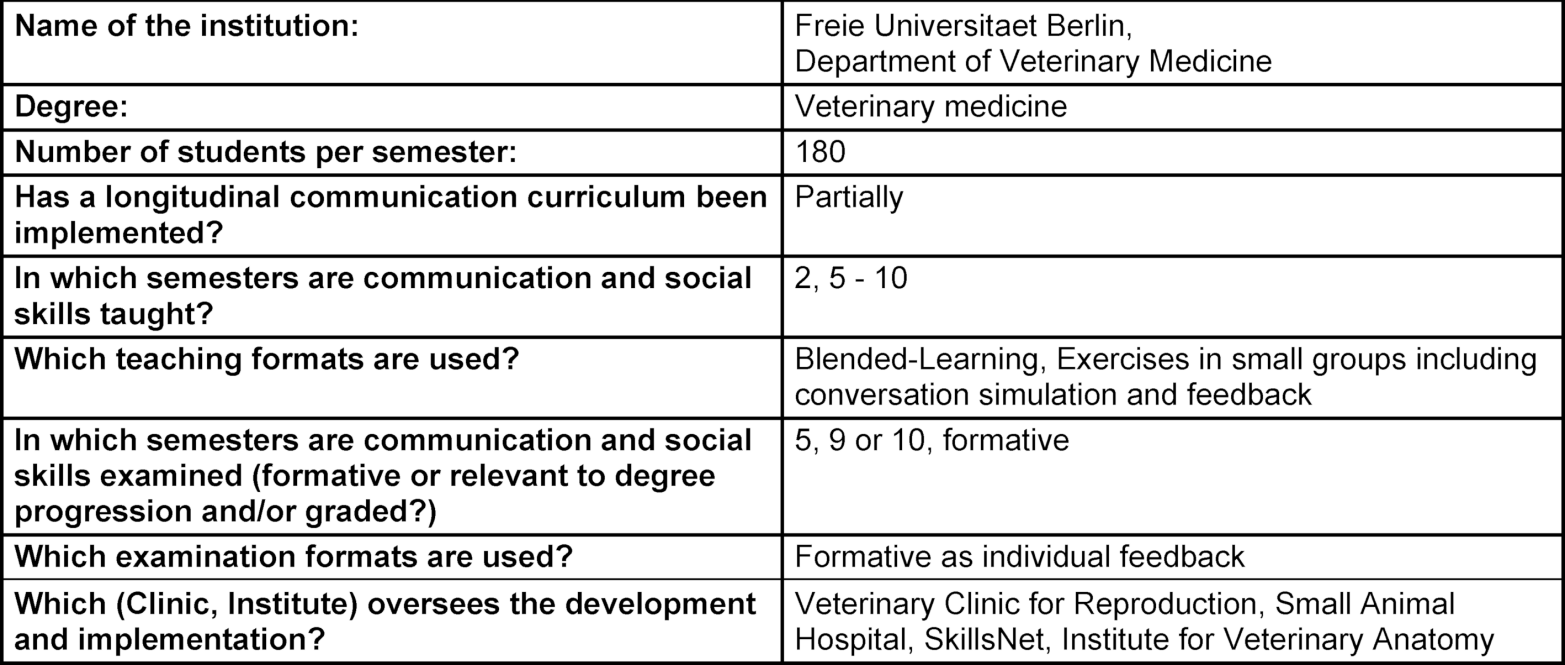
Questionnaire Freie Universitaet Berlin, answered by A. Pohl and M. Bahramsoltani

**Table 2:**
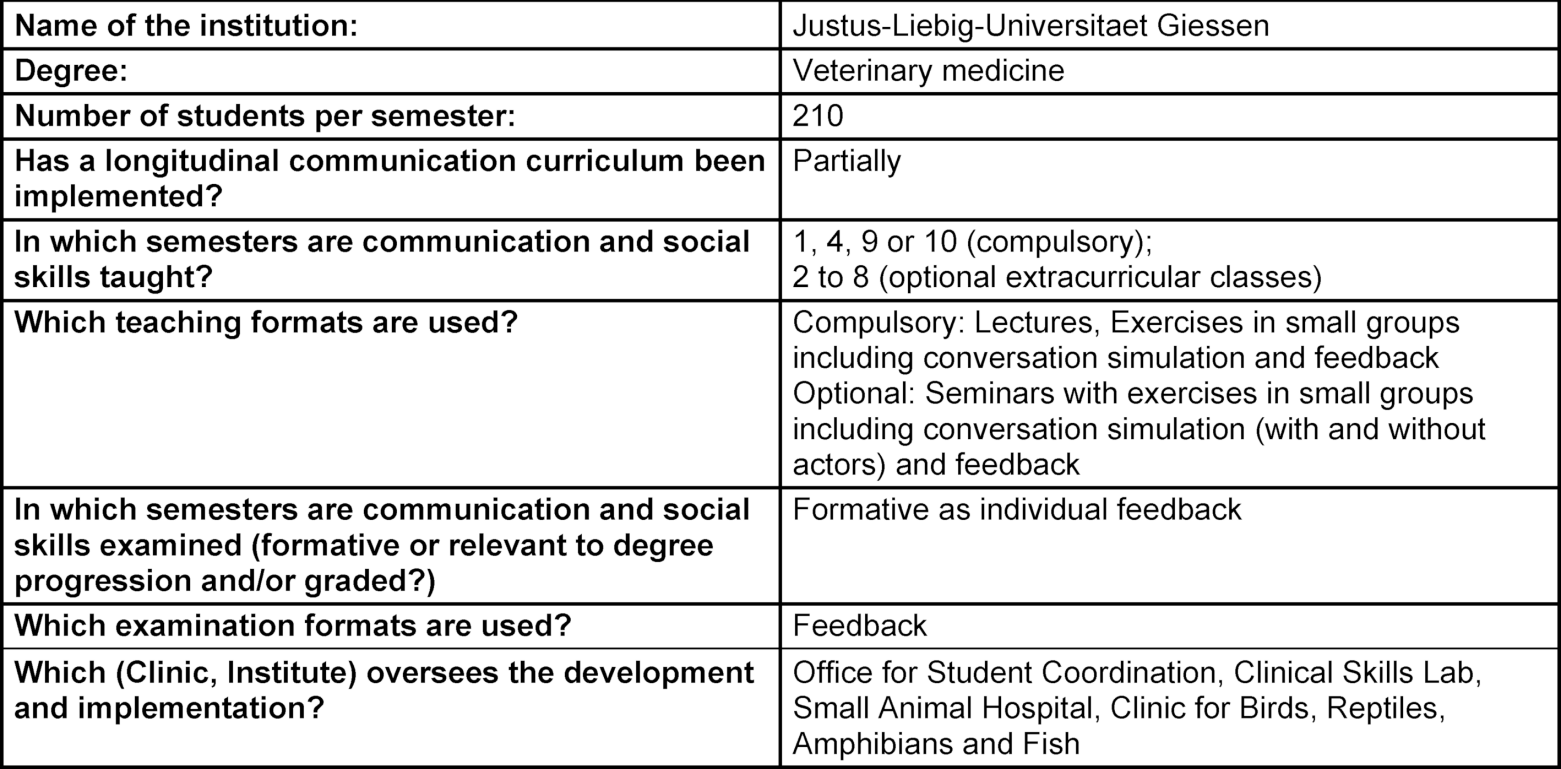
Questionnaire Justus-Liebig-Universitaet Gießen, answered by B. Pfeiffer-Morhenn

**Table 3:**
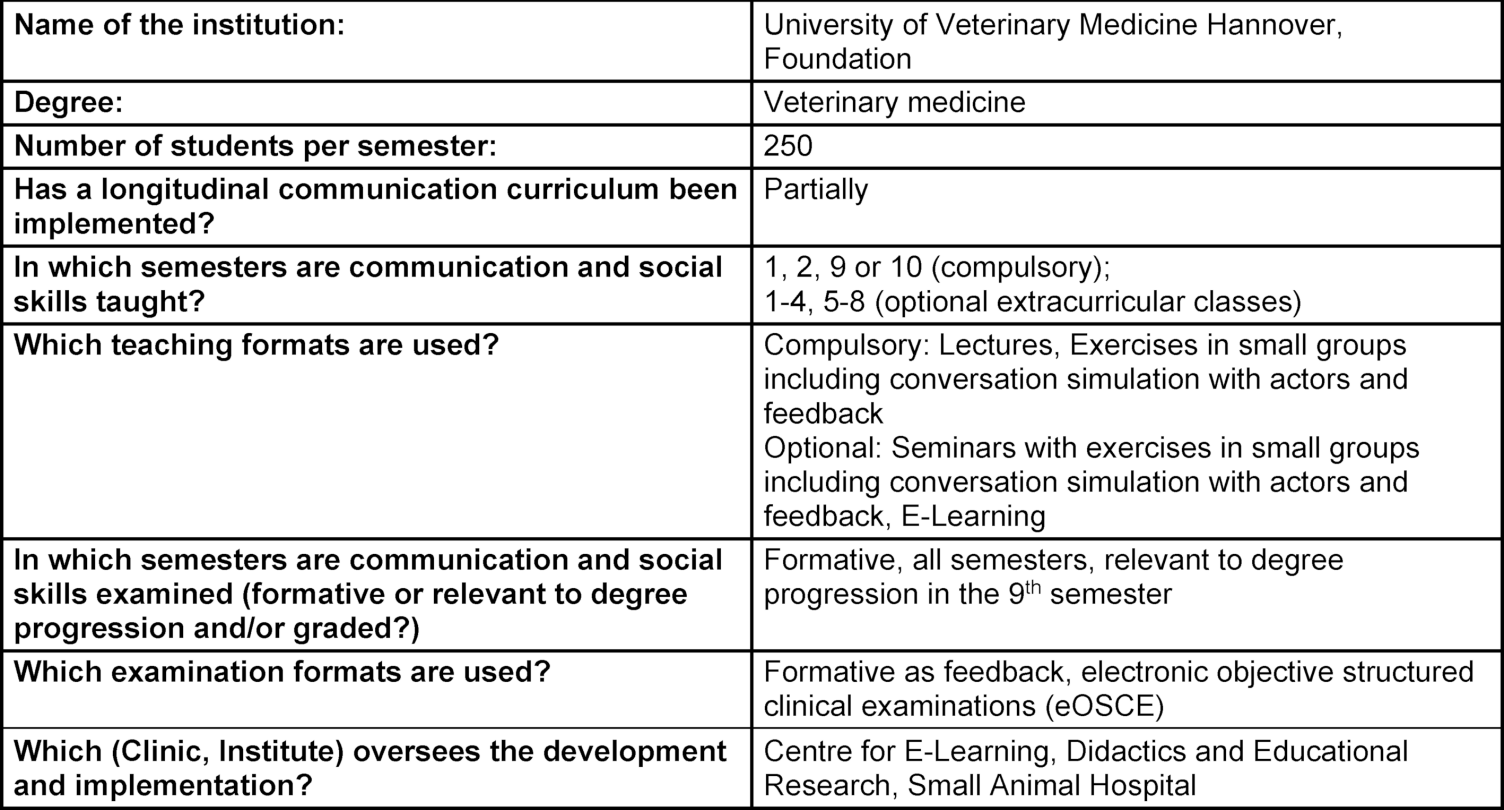
Questionnaire University of Veterinary Medicine Hannover, Foundation, answered by C. Kleinsorgen and S. Wissing

**Table 4:**
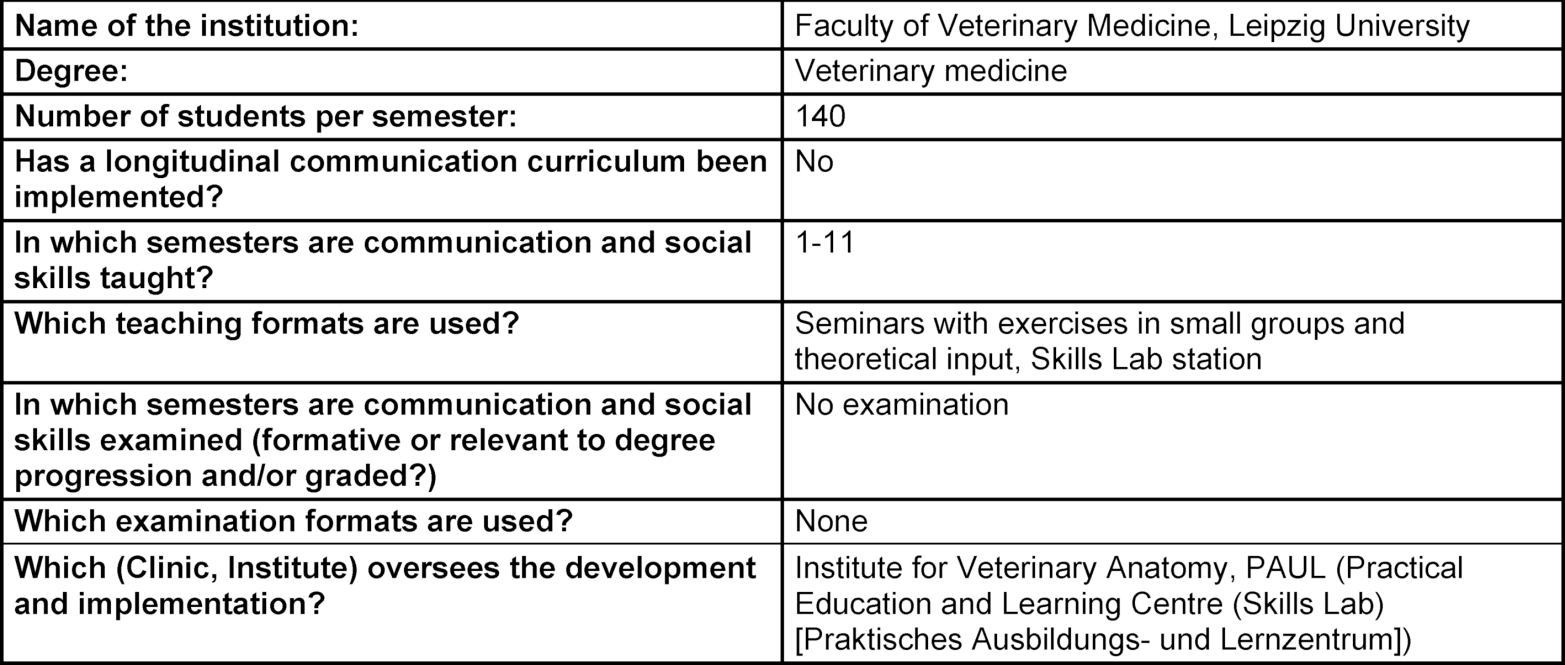
Questionnaire University of Leipzig, answered by D. Bernigau

**Table 5:**
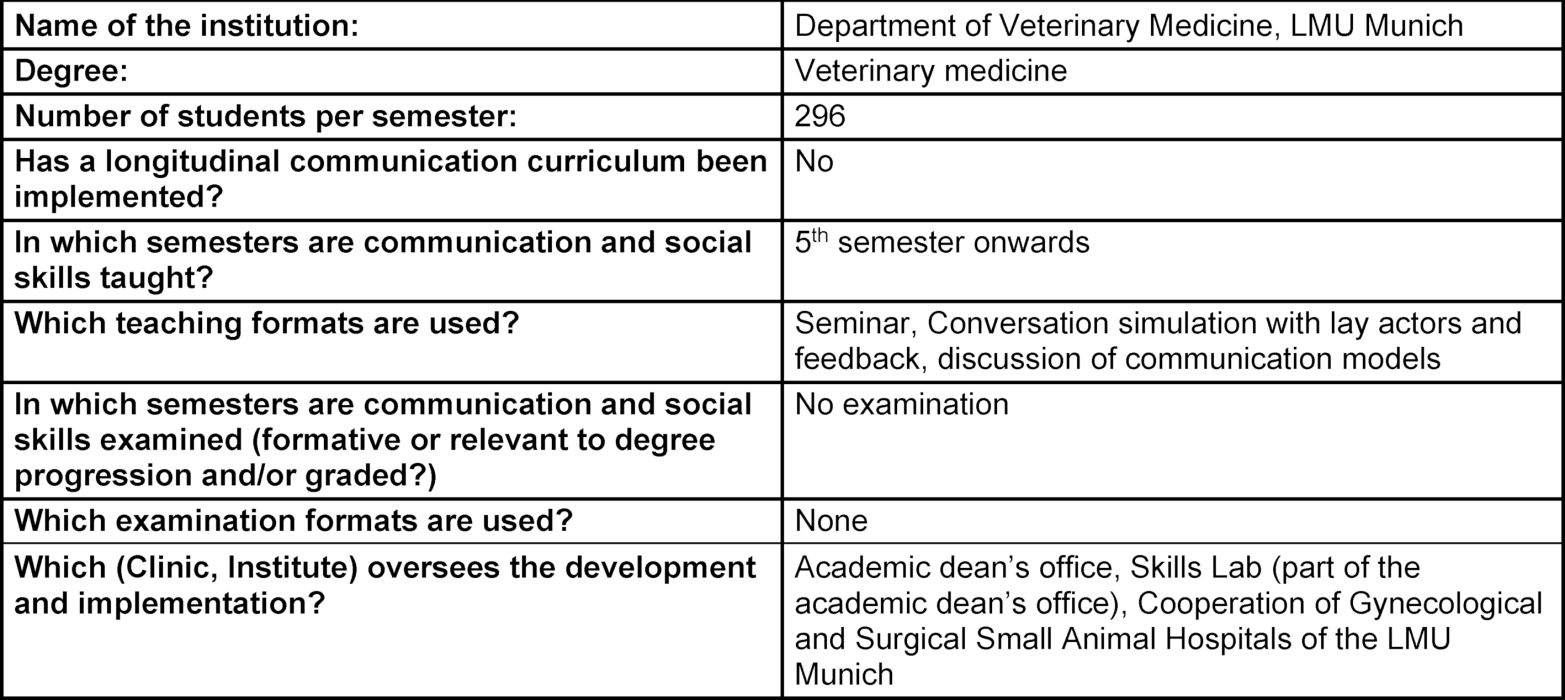
Questionnaire Ludwig-Maximilians-University Munich, answered by C. Beitz-Radzio
