# Supplementary material for: Integration and potential of teaching communication skills in the study of veterinary medicine in Germany
Source: GMS J Med Educ. 2021 Mar 15;38(3):Doc53. doi: 10.3205/zma001449 (PMC7994865; doi:10.3205/zma001449)
Supplement: Communication education at the five German veterinary education institutions; results from a written questionnaire among teachers from the respective institutions [file JME-38-3-53-s-001.pdf]

Attachment 1: Communication education at the five German veterinary education institutions; results from a written questionnaire among teachers from the respective institutions

Place	Teaching format	Teaching content	Learning goals*	Participants	Duration	Examination
Berlin	Blended-Learning during cross-section classes	Theoretical models, application exercises	1 a; 2 a, b; 3a; 4a; 6 a, b; 8 a; 9 a, b	2. semester, compulsory	14 h	No
	Communication Day during the project week	Theoretical basics, conversation simulation	1 a, b, c; 2 a, b, c; 3 a, b, c; 4 a, b, c; 5 a, b, c; 6 a, b; 8 a; 9 a, b, c	5. semester, optional	6 h	Formative
	Extracurricular class	Theoretical basics, practical exercises, conversation simulation	1 a, b, c; 2 a, b, c; 3 a, b, c; 4 a, b, c; 5 a, b, c; 6 a, b; 7 c; 8 a; 9 a, b, c	5.-8. semester, optional,<br>10-15 participants, irregular	14 h	Formative
	Exercise during the practical year	Theoretical basics, practical exercises,	1 a, b; 2 a, b; 3 a, b; 4 a, b; 8 a; 9 a, b, c	9.-10. semester, compulsory	2 h	No
Leipzig	Extracurricular class	Theoretical basics, practical exercises, conversation simulation	1 a, b; 2 a, b; 3 a, b, c; 5 a, b; c; 6 a, b, c; 8 a, b: 9 a, b	All semesters, optional,<br>30 participants	14 h	No
	Exercise, Skills Lab station on communication	Multiple case presentations as role play exercises in German and English	1 a, b, c; 2 a, b, c; 3 a, b, c; 4 a, b, c	All semesters, optional		No
Hannover	Lecture as part of veterinary professional studies course	Theoretical models, application exercises	1 a; 2 a; 5 a; 8 a; 9 a	1.-2. semester, compulsory	4 h	No
	Exercise during the practical year for students specializing in small animals and equines (currently under construction)	Theoretical basics, practical exercises, conversation simulation	1 a, b, c; 2 a, b, c; 3 a, b, c; 4 a, b, c; 5 a, b, c; 6 a, b; 7 a, b, c; 8 a, b, c; 9 a, b, c	9.-10. semester, compulsory	6 h (min.)	Formative
	Extracurricular class	Basics, theoretical models, application exercises	1 a, b; 2 a, b; 3 a; 4 a; 5 a, b, c; 8 a, b; 9 a, b, c	1.-4. semester, optional,<br>16 participants	4 h	Formative
	Extracurricular class	Conversation simulation (difficult situations, obtaining anamneses)	1 a, b, c; 2 a, b, c; 3 a, b, c; 4 a, b, c; 5 a, b, c; 6 a, b; 7 a, b, c; 8 a, b, c; 9 a, b, c	5.-8. semester, optional,<br>8 participants	10 h	Formative
	Lecture as part of the veterinary professional studies course, exercise during the practical year for students specialized on small animals, equines, and small ruminants	How to address euthanasia	1 a, b, c; 2 a, b, c; 3 a, b, c; 4 a, b, c; 5 a, b, c; 6 a, b; 7 a, b, c; 8 a, b, c; 9 a, b, c	5.-8. semester, optional,<br>8 participants	6 h	Formative

Attachment 1 to: Pohl, A, Klass LC, Kleinsorgen C, Bernigau D, Pfeiffer-Morhenn B, Arnhold S, Dilly M, Beitz-Radzio C, Wissing S, Vogt L, Bahmramsoltani M. *Integration and potential of teaching communication skills in the study of veterinary medicine in Germany.* GMS J Med Educ. 2021;38(3):Doc53. DOI: 10.3205/zma001449

	Extracurricular class	Theoretical models, practical exercises, conversation simulation (Giving bad news, addressing euthanasia)	1 a, b, c; 2 a, b, c; 3 a, b, c; 4 a, b, c; 5 a, b, c; 6 a, b; 7 a, b, c; 8 a, b, c; 9 a, b, c	5.-8. semester, optional,<br>16 participants	6 h	Formative
	Extracurricular class	Scientific presentations and job applications	2 a, b, c; 5 a, b, c; 8 a, b, c; 9 a, b, c	5.-8. semester, optional,<br>8 participants	10 h	Formative
	Advanced training	conversation simulation (difficult situations, obtaining anamneses)	1 a, b, c; 2 a, b, c; 3 a, b, c; 4 a, b, c; 5 a, b, c; 6 a, b; 7 a, b, c; 8 a, b, c; 9 a, b, c	Student assistants and interns from the small animal hospital, 30 participants	3 h	Formative
	Exercise, Skills Lab station on communication (under construction)	conversation simulation (obtaining anamneses)	1 a, b, c; 2 a, b, c; 3 a, b, c; 4 a, b, c; 5 a, b, c; 6 a, b; 7 a, b, c; 8 a, b, c; 9 a, b, c	All semesters, optional	2 h	No
	E-Learning	Various modules, theoretical models, application exercises	2 a, b; 3 a, b; 4 a, b; 5 a, b, c; 6 a, b; 7 a, b; 8 a, b; 9 a, b	All semesters, optional	30-45 min each	No
Giessen	Lecture as part of the veterinary professional studies course	Theoretical basics	1 a, b; 2 a, b; 3 a, b; 4 a; 5 a, b; 7 a; 8 a; 9 a, b	1. semester, compulsory	4 h	No
	Exercise during the propaedeutics class, Skills Lab	Theoretical basics, obtaining anamneses	1 a, b, c; 2 a, b, c; 3 a, b, c; 4 a, b, c; 5 a, b, c; 6 a, b; 9 a, b, c	4. semester, compulsory	10 h	Formative
	Exercise during the practical year, Skills Lab	Theoretical basics, obtaining anamneses	1 a, b, b; 2 a, b, c; 3 a, b, c; 4 a, b, c; 5 a, b, c; 6 a, b; 9 a, b, c	9.-10. semester, compulsory	10 h	Formative
	Extracurricular class	Theoretical basics, conversation simulation	1 a, b, c; 2 a, b, c; 3 a, b, c; 4 a, b, c; 5 a, b, c; 6 a, b, c; 9 a, b, c	2.-7. semester, optional,<br>5-30 participants	14 h	Formative
	Extracurricular class	Communication in emergency medicine	1 a, b, c; 2 a, b, c; 3 a, b, c; 4 a, b, c; 5 c; 6 a, b, c; 7 a, b, c; 8 a; 9 a	5.-8. semester, optional,<br>12 participants from veterinary medicine, 12 participants from human medicine, 12 participants from psychology	14 h	Formative
	Extracurricular class	Conflicts and stressful situations	1 c; 2 c; 3 c; 4 c; 5 c; 6 c; 7 c; 8 b; 9 a	6. and 8. semester, optional,<br>5-15 participants	16 h	Formative
	Extracurricular class	Theoretical basics, conversation simulation	1 a, b, c; 2 a, b, c; 3 a, b, c; 4 a, b, c; 5 a, b, c; 6 a, b, c; 7 a, b, c; 8 a, b, c; 9 a, b, c	6.-9. semester, optional,<br>5-15 participants	14 h	Formative

Munich	Exercise during the practical year for students specializing in small animals and equines	Theoretical basics, conversation simulation	1 a, b; 2 a; 3 a, b, c; 4 a, b; 7 a; 8 a, b; 9 a	9.-10. semester, compulsory	2 h	No
	Extracurricular class	Theoretical basics, conversation simulation, strategies for difficult conversations	1 a, b, c; 2 a, b, c; 3 a, b, c; 4 a, b, c; 5 a, b, c; 6 a, b; 7 a, b, c; 8 a, b; 9 a, b, c	Optional	14 h	Formative
	Exercise, Skills Lab station on communication	Theoretical basics, simulation of patient owner communication	1 a, b; 2 a, b; 3 a, b; 4 a, b; 5 a; 6 a; 7 a, b; 8 a, b; 9 a, b	All semesters, optional		No
	Advanced training	Theoretical basics, simulation of emergency situations	1 a, b, c; 2 a; 3 a, b; 4 a, b; 6 a, b; 7 a, b, c; 8 a; 9 a, b, c	Hospital staff, externs, compulsory		No
